# Combined Anti-Inflammatory Effects of Curcumin and Evodiamine: In Vitro Synergy, Docking, and Molecular Orbital Insights

**DOI:** 10.3390/ijms27093834

**Published:** 2026-04-25

**Authors:** Sarin Tadtong, Kanyanat Atiwanitchakul, Muna Moohammad, Chuda Chittasupho, Chatchapong Tangjidapichai, Weerasak Samee

**Affiliations:** 1 Faculty of Pharmacy, Srinakharinwirot University, Nakhon Nayok 26120, Thailand; sarin@g.swu.ac.th (S.T.); kanyanat.atiwa@g.swu.ac.th (K.A.); muna.moo@g.swu.ac.th (M.M.); chatchapong@g.swu.ac.th (C.T.); 2 Faculty of Pharmacy, Chiang Mai University, Chiang Mai 50200, Thailand; chuda.c@cmu.ac.th

**Keywords:** curcumin, evodiamine, synergism, anti-inflammatory, docking, molecular orbital, HPLC

## Abstract

Combining plant-derived bioactives could produce effective anti-inflammatory interventions for myofascial inflammation. This study evaluated in vitro synergy and computational mechanisms of curcumin–evodiamine activity against TNF-α, IL-1β, iNOS and COX-2, with frontier molecular orbital analysis to inform putative mechanisms. Evodiamine and curcumin were identified/quantified by HPLC–PDA and LC–MS (λmax 226 nm and 426 nm; RT 8.61 and 9.53 min; [M−H]^−^ *m*/*z* 302.2 and 367.2). Purities were 98.08 ± 1.92% and 98.04 ± 1.86%. Noncytotoxic concentrations in RAW264.7 cells were determined, then LPS-stimulated cells were treated with evodiamine (0.01 µM), curcumin (0.01 µM) and a 1:1 mixture (0.001 µM). Molecular docking against TNF-α, IL-1β, iNOS and COX-2 and HOMO–LUMO calculations were performed. Curcumin and the combination significantly reduced TNF-α and NO; curcumin and the combination reduced IL-1β, whereas evodiamine alone showed limited effects. Docking predicted stronger binding for curcumin and evodiamine than ibuprofen across targets (e.g., curcumin ΔG −10.18 kcal·mol^−1^ for TNF-α; evodiamine ΔG −10.02 kcal·mol^−1^ for COX-2). Frontier orbital energies indicated differing electronic profiles (ibuprofen ΔE 8.62 eV; evodiamine 9.65 eV; curcumin 9.89 eV), suggesting complementary reactivity. The curcumin–evodiamine combination exhibits in vitro anti-inflammatory activity with supportive docking and orbital data, providing mechanistic rationale for further development.

## 1. Introduction

Myofascial inflammation (myositis) results from prolonged or repetitive muscle use and is characterized by deep, radiating muscle pain, myofascial trigger points, fibrosis, and muscle stiffness. The condition is prevalent among manual laborers and working-age adults and may present as acute or chronic myositis, imposing substantial functional impairment and socioeconomic burden [1]. At the cellular level, muscle injury provokes the release of damage-associated molecular patterns (DAMPs), which engage Toll-like receptors on skeletal muscle fibers, infiltrating macrophages, dendritic cells, endothelial cells, and stromal cells. This innate immune activation establishes a proinflammatory microenvironment rich in type I interferons, TNF-α, IL-1 family cytokines, IL-12, and IFN-γ, amplifying local inflammation and promoting microvascular injury and hypoxia. Activation of TNF signaling stimulates the IκB kinase (IKK) complex and downstream NF-κB translocation (RelA/p50), thereby upregulating proinflammatory mediators including TNF-α, IL-1β, and inducible nitric oxide synthase (iNOS), factors implicated in impaired myogenic programs, muscle fiber damage, and weakness [2].

Current pharmacological management of inflammatory muscle pain principally relies on nonsteroidal anti-inflammatory drugs (NSAIDs), which inhibit cyclooxygenase (COX) enzymes and reduce synthesis of prostaglandins and other inflammatory mediators. While effective, NSAID therapy is associated with a range of adverse effects—gastrointestinal, hepatic, cardiovascular, respiratory, and dermatologic—that limit long-term use in many patients. Consequently, there is considerable interest in complementary and alternative therapies with improved safety profiles [3,4,5,6,7,8].

Traditional medicinal plants offer a rich source of anti-inflammatory compounds. *Curcuma longa* (turmeric) and *Evodia rutaecarpa* (Wu Chu Yu) have long histories of use across South, Southeast and East Asia for treatment of pain and inflammatory disorders. Curcumin, the principal curcuminoid in turmeric, exhibits anti-bacterial, antioxidant, COX-2 inhibitory activity and suppresses TNF-α signaling; clinical studies report curcumin’s efficacy for osteoarthritis pain at both low (<1000 mg/day) and high (>1000 mg/day) doses, with an adverse-event profile more favorable than that of NSAIDs. Mechanistically, curcumin inhibits NF-κB activation in chondrocytes, reduces apoptosis, downregulates matrix metalloproteinases and proteoglycan degradation, and attenuates exercise-induced muscle damage by lowering serum creatine kinase and proinflammatory cytokines such as TNF-α, IL-6, and IL-8 [9,10,11,12,13,14,15,16].

Evodiamine, an alkaloid isolated from *E. rutaecarpa*, is another bioactive compound with documented anti-inflammatory effects. Phytochemical analyses identify evodiamine as a predominant constituent in *E. rutaecarpa* extracts [17], and in vitro studies demonstrate that evodiamine inhibits COX-2–dependent prostaglandin E2 synthesis, suppresses NF-κB activation, and reduces production of nitric oxide, IL-6, TNF-α, and PGE2 in lipopolysaccharide-stimulated macrophage models at micromolar concentrations. These activities indicate that evodiamine modulates key signaling pathways central to myofascial inflammation, including TNF-α and IL-1β signaling cascades [18,19,20].

Although curcumin and evodiamine individually target overlapping inflammatory mediators and signaling nodes (COX-2, NF-κB, TNF-α, IL-1β, and iNOS), their combined effects on muscle inflammation have not been thoroughly investigated. Synergistic interactions between phytochemicals can enhance potency, broaden mechanistic coverage, and reduce required dosages, potentially improving therapeutic indices relative to single-agent use. Moreover, integrating in vitro functional assays with in silico molecular docking against TNF-α, IL-1β, and nitric oxide synthase may elucidate complementary binding interactions and mechanistic bases for observed synergy [21,22,23].

The anti-inflammatory activity of small molecules is intimately linked to their electronic structure, particularly the energies of the highest occupied molecular orbital (HOMO) and the lowest unoccupied molecular orbital (LUMO). These frontier orbitals modulate molecular reactivity, redox properties, and the propensity to engage with biological targets. A reduced HOMO–LUMO energy gap typically correlates with increased chemical reactivity and enhanced anti-inflammatory potential, as narrower gaps often confer greater molecular polarization and reactivity that facilitate interactions with inflammation-related enzymes such as cyclooxygenase-2 (COX-2). Elevated HOMO energies indicate an increased propensity for electron donation (antioxidant behavior), whereas low LUMO energies reflect a heightened capacity for electron acceptance; both characteristics can disrupt proinflammatory signaling cascades. Potent anti-inflammatory agents commonly display small HOMO–LUMO gaps and elevated electrophilicity indices, attributes that promote antioxidant activity and mitigate oxidative stress-mediated inflammation. Consequently, the HOMO–LUMO gap is often directly associated with anti-inflammatory efficacy, with smaller gaps frequently corresponding to stronger suppression of nitric oxide production and reduction in proinflammatory cytokine expression [24,25,26].

Therefore, this study aims to evaluate the combined anti-inflammatory effects of curcumin and evodiamine using in vitro inflammation models and molecular docking analyses directed at TNF-α, IL-1β, iNOS, and COX-2. In addition, frontier molecular orbitals will be calculated to inform putative anti-inflammatory mechanisms. We hypothesize that the curcumin–evodiamine combination will exert synergistic inhibition of proinflammatory cytokine production, COX-2 and iNOS activity, and NF-κB signaling (TNF-α, IL-1β), thereby providing a mechanistic rationale for the development of safer, plant-derived interventions for myofascial inflammation.


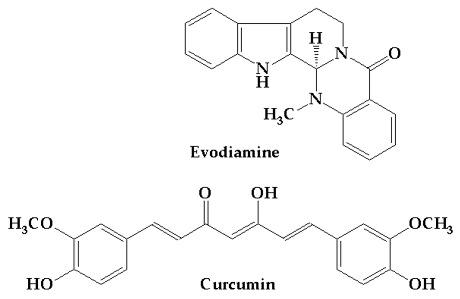


## 2. Results

### 2.1. Identification and Quantification of Curcumin and Evodiamine Raw Materials

**Figure 1:**
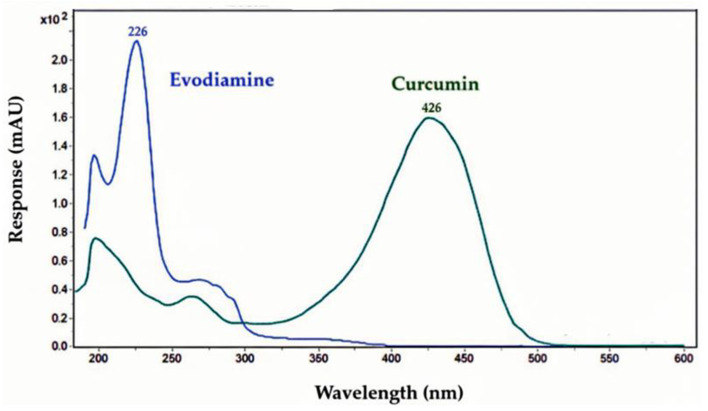
UV–VIS spectra of evodiamine and curcumin. The maximum absorption wavelengths (λmax) were 226 nm for evodiamine and 426 nm for curcumin.

**Figure 2:**
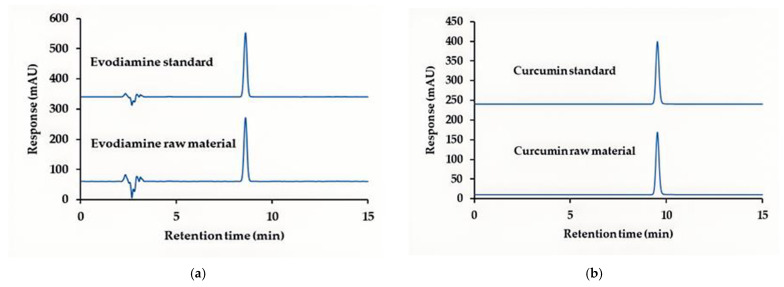
HPLC retention-time comparison with reference standards: (**a**) evodiamine (8.61 min) detected at 226 nm; (**b**) curcumin (9.53 min) detected at 426 nm.

To identify curcumin and evodiamine in raw materials, high-performance liquid chromatography with photodiode array detection (HPLC–PDA) coupled with mass spectrometry (LC–MS) was employed. The maximum UV–Vis absorption wavelengths (λmax) for evodiamine and curcumin were 226 nm and 426 nm, respectively (Figure 1). HPLC–PDA analysis (Figure 2a,b) gave retention times of 8.61 min for evodiamine and 9.53 min for curcumin. LC–MS in negative ion mode produced fragment ions at *m*/*z* 302.2 and 367.2, consistent with the deprotonated molecular ions ([M−H]−) of evodiamine (M = 303.36 g·mol^−1^) and curcumin (M = 368.38 g·mol^−1^), and these identifications were used for subsequent quantification.

Percentage purity of the raw materials was determined using a single-point calibration method by comparing the HPLC peak area of each sample with that of the corresponding reference standard. As shown in Table 1, the purities of evodiamine and curcumin were 98.08 ± 1.92% and 98.04 ± 1.86%, respectively.

### 2.2. Effect of Evodiamine and Curcumin on the Viability of RAW264.7 Cells

**Figure 3:**
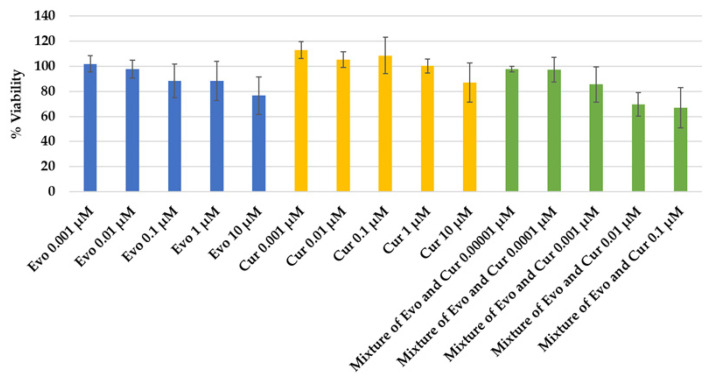
Cell viability of RAW264.7 cells treated with evodiamine (Evo), curcumin (Cur), and their 1:1 combination.

**Figure 4:**
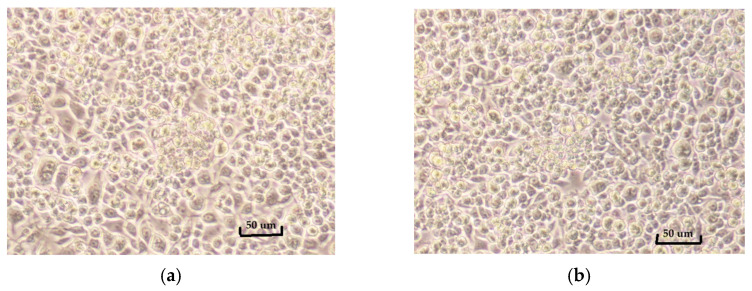
Morphology of RAW264.7 cells: (**a**) untreated control; (**b**) cells treated with 10 µM curcumin (86.83% viability).

Cell viability assays in RAW264.7 cells were used to identify non-cytotoxic concentrations of evodiamine, curcumin, and their 1:1 combination for subsequent anti-inflammatory testing. As summarized in Figure 3, five concentrations of each test material were evaluated (*n* = 3). The lowest concentrations that maintained >80% viability and were selected for downstream assays were: 1 µM of evodiamine, 10 µM of curcumin, and 0.001 µM of the evodiamine–curcumin (1:1) mixture. Individual compounds exhibited generally higher viability across concentrations than the combination. Representative values include evodiamine (0.001–10 µM: 101.7 ± 6.4 to 76.7 ± 14.9% viability), curcumin (0.001–10 µM: 112.9 ± 6.8 to 86.8 ± 15.5% viability), and the 1:1 mixture (0.00001–0.1 µM: 97.7 ± 2.2 to 66.7 ± 16.0% viability). As shown in Figure 4, the morphology of untreated RAW264.7 cells and cells maintaining >80% viability is quite similar.

The viability data demonstrate concentration-dependent effects for all test conditions and indicate that the evodiamine–curcumin combination is more cytotoxic to RAW264.7 cells than either compound alone at equivalent nominal concentrations. Curcumin exhibited the highest tolerability, with viability >100% at lower concentrations and remaining above 80% up to 10 µM. Evodiamine showed a modest decline in viability at higher concentrations, crossing below 80% at 10 µM. Notably, the 1:1 mixture produced reduced viability at much lower nominal concentrations (viability <80% at 0.01 µM and 0.1 µM), such that a mixture concentration of 0.001 µM was the highest tested that consistently preserved >80% viability.

Possible explanations for the increased toxicity of the combination include pharmacodynamic interaction (additive or synergistic cytotoxicity), altered cellular uptake or solubility when combined, or matrix/solvent effects at the tested concentrations. The relatively large standard deviations observed for some concentrations, particularly at higher doses, suggest variability that should be addressed with larger sample sizes.

### 2.3. Anti-Inflammatory Effects of Evodiamine, Curcumin, and Their Combination on LPS-Induced RAW264.7 Cells

To compare the anti-inflammatory effects of evodiamine, curcumin, and their combination, RAW264.7 cells were stimulated with LPS (µg·mL^−1^) and treated with evodiamine (0.01 μM), curcumin (0.01 μM), and the evodiamine–curcumin (1:1) mixture (0.001 μM).

#### 2.3.1. Effects of Evodiamine, Curcumin, and Their Combination on TNF-α Levels in LPS-Stimulated RAW 264.7 Cells

As shown in Table 2, TNF-α concentrations (mean ± SD, ng·mL^−1^) were 1.99 ± 0.19 for 0.5% DMSO (solvent control), 4.81 ± 0.90 for 1 µg·mL^−1^ LPS (negative control), 3.91 ± 0.37 for 50 µM ibuprofen (positive control), 5.56 ± 0.18 for evodiamine 0.01 µM, 3602.60 ± 282.78 for curcumin 0.01 µM, and 3.12 ± 0.38 for the evodiamine–curcumin (1:1) mixture at 0.001 µM. Relative to the LPS-treated negative control, 50 µM ibuprofen, curcumin (0.01 µM), and the evodiamine–curcumin mixture (0.001 µM) produced statistically significant reductions in TNF-α, whereas evodiamine (0.01 µM) did not. The data indicate that curcumin at 0.01 µM and the low-concentration evodiamine–curcumin mixture attenuate LPS-induced TNF-α release in RAW264.7 cells, consistent with reported anti-inflammatory activity of curcumin and potential cooperative effects in combinations. Ibuprofen’s partial suppression of TNF-α corroborates its expected anti-inflammatory profile under these conditions. In contrast, evodiamine at 0.01 µM yielded a higher TNF-α level than LPS alone, suggesting that, at this concentration, evodiamine may lack anti-inflammatory efficacy in this model or could exert pro-inflammatory or cytotoxic effects that secondarily enhance cytokine release. The markedly lower effective concentration of the evodiamine–curcumin mixture (0.001 µM) compared with the individual compounds raises the possibility of synergistic interaction, dose-dependent modulation, or altered bioavailability when combined; however, the mechanism remains unclear.

#### 2.3.2. Effects of Evodiamine, Curcumin, and Their Combination on IL-1β Levels in LPS-Stimulated RAW 264.7 Cells

As shown in Table 2, IL-1β concentrations (mean ± SD, pg·mL^−1^) were: 0.00 for 0.5% DMSO (solvent control), 6.12 ± 9.42 for 1 µg·mL^−1^ LPS (negative control), 0.00 for 50 µM ibuprofen (positive control), 2.68 ± 4.22 for evodiamine 0.01 µM, 0.00 for curcumin 0.01 µM, and 0.00 for the evodiamine–curcumin (1:1) mixture at 0.001 µM. Compared with the LPS-treated negative control, both 50 µM ibuprofen and curcumin (0.01 µM) produced statistically significant reductions in IL-1β, whereas evodiamine (0.01 µM) did not. The IL-1β data indicate that curcumin at 0.01 µM and ibuprofen at 50 µM effectively suppress LPS-induced IL-1β release in RAW264.7 cells under the conditions tested, consistent with their reported anti-inflammatory properties. The evodiamine result (2.68 ± 4.22 pg·mL^−1^) did not differ significantly from the LPS control, suggesting that at 0.01 µM evodiamine lacks a measurable inhibitory effect on IL-1β production in this model. The evodiamine–curcumin mixture (0.001 µM) yielded undetectable IL-1β, which may reflect effective suppression at the tested combination concentration or assay sensitivity limits. Several considerations arise from these findings. First, the large SD for the LPS control (9.42 pg·mL^−1^) and low absolute cytokine values indicate high variability and measurements near the assay detection limit, which reduces confidence in small differences. Second, the differential effects between individual compounds and the combination could reflect additive or synergistic interactions, altered cellular uptake, or concentration-dependent modulation of inflammatory signaling. Third, the absence of detectable IL-1β in some groups may be due to true suppression or technical floor effects of the assay.

#### 2.3.3. Effects of Evodiamine, Curcumin, and Their Combination on NO Levels in LPS-Stimulated RAW 264.7 Cells

As shown in Table 2, nitric oxide (NO) levels (µM) were: 10.00 for 0.5% DMSO (solvent control), 13.77 for 1 µg·mL^−1^ LPS (negative control), 6.33 for 50 µM ibuprofen (positive control), 15.18 for evodiamine 0.01 µM, 7.24 for curcumin 0.01 µM, and 6.24 for the evodiamine–curcumin (1:1) mixture at 0.001 µM. Relative to the LPS-treated negative control, 50 µM ibuprofen, curcumin (0.01 µM), and the evodiamine–curcumin mixture (0.001 µM) produced statistically significant reductions in NO, whereas 0.5% DMSO and evodiamine (0.01 µM) did not.

The data indicate that curcumin and the evodiamine–curcumin combination effectively suppressed LPS-induced NO production in RAW264.7 cells, achieving reductions comparable to or exceeding that of the positive control (ibuprofen). In contrast, evodiamine alone at 0.01 µM did not attenuate NO and, in fact, yielded higher NO than the LPS control, suggesting that at this concentration evodiamine lacks inhibitory activity on inducible nitric oxide synthase (iNOS) expression or activity in this model; it may also exert pro-inflammatory or cytotoxic effects that indirectly influence NO production. The pronounced effect of the 1:1 combination at a markedly lower nominal concentration (0.001 µM) suggests possible potentiation or altered bioavailability when compounds are combined. Potential mechanisms include synergistic modulation of NF-κB/MAPK signaling pathways, direct scavenging of reactive nitrogen species by curcumin, or enhanced cellular uptake/retention of active moieties in the mixture. However, differences in nominal concentrations and the absence of mechanistic readouts preclude firm conclusions regarding synergy.

### 2.4. Docking Analysis: Curcumin, Evodiamine and Ibuprofen Interactions with TNF-α, IL-1β, iNOS, and COX-2

As shown in Table 3, more negative binding energies (ΔG) and smaller predicted inhibition constant (Ki) values indicate stronger ligand–target interactions. Across the four examined inflammatory targets (TNF-α, IL-1β, iNOS, and COX-2), curcumin and evodiamine exhibited substantially stronger predicted binding than ibuprofen.

**Figure 5:**
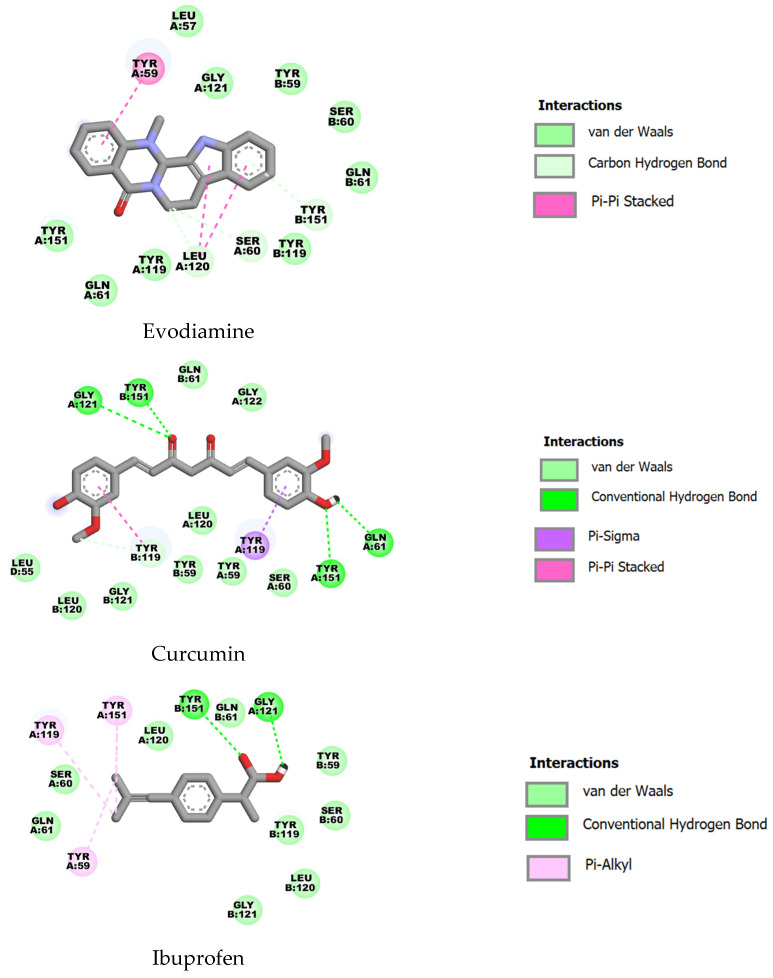
Two-dimensional representations of the binding interactions of evodiamine, curcumin and ibuprofen with TNF-α (PDB ID: 2AZ5).

For TNF-α (PDB ID: 2AZ5), curcumin showed the highest predicted affinity (ΔG = −10.18 kcal·mol^−1^, Ki ≈ 0.03 µM), followed by evodiamine (ΔG = −8.35 kcal·mol^−1^, Ki ≈ 0.76 µM) and ibuprofen (ΔG = −6.00 kcal·mol^−1^, Ki ≈ 39.7 µM). All three ligands contacted a common set of residues (including TYR59, SER60, GLY61, TYR119, LEU120, GLY121 and TYR151), consistent with binding to an overlapping pocket; the markedly lower energy and Ki for curcumin suggest more favorable interactions within this site (Figure 5).

**Figure 6:**
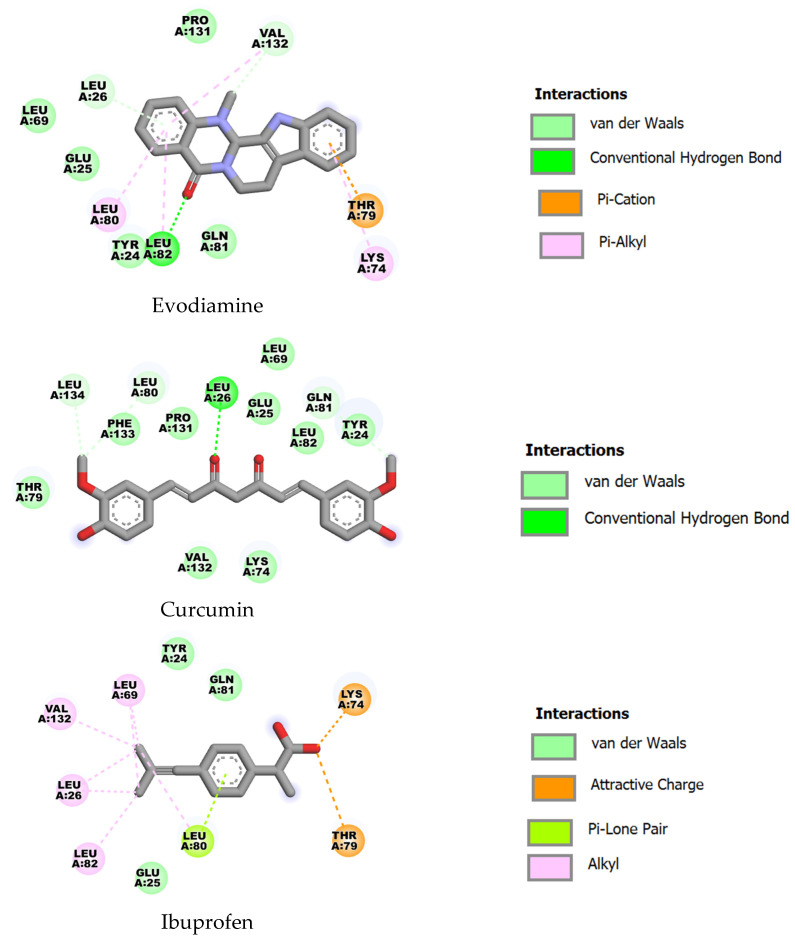
Two-dimensional representations of the binding interactions of evodiamine, curcumin and ibuprofen with IL-1β (PDB ID: 5R8Q).

With IL-1β (PDB ID: 5R8Q), curcumin again displayed the strongest predicted binding (ΔG = −8.73 kcal·mol^−1^, Ki ≈ 0.40 µM), followed by evodiamine (ΔG = −7.72 kcal·mol^−1^, Ki ≈ 2.19 µM) and ibuprofen (ΔG = −6.24 kcal·mol^−1^, Ki ≈ 26.9 µM). The ligands share several contact residues (e.g., TYR24, GLU25, LEU26, LEU69, LYS74, THR79, LEU80, GLN81, LEU82); curcumin’s engagement of additional residues such as PHE133 and LEU134 are likely to contribute to its superior affinity (Figure 6).

**Figure 7:**
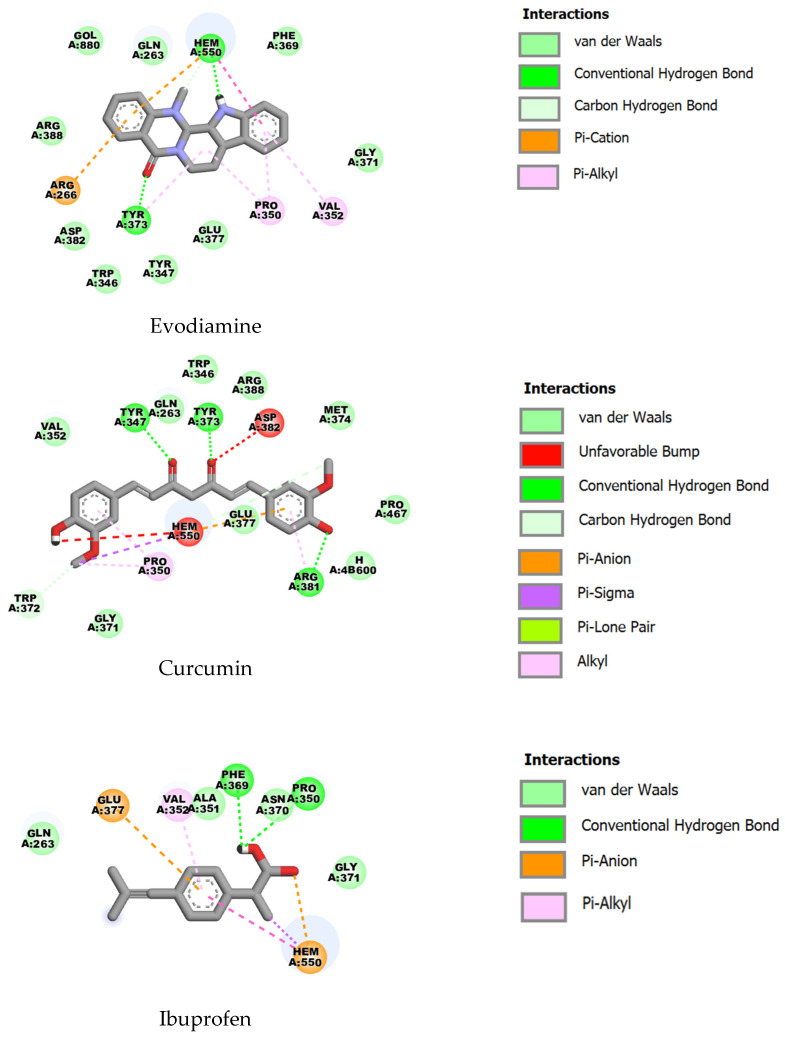
Two-dimensional representations of the binding interactions of evodiamine, curcumin and ibuprofen with iNOS (PDB ID: 4CX7).

For inducible nitric oxide synthase (iNOS; PDB ID: 4CX7), evodiamine (ΔG = −8.95 kcal·mol^−1^, Ki ≈ 0.27 µM) and curcumin (ΔG = −8.87 kcal·mol^−1^, Ki ≈ 0.31 µM) exhibited similarly strong predicted binding, whereas ibuprofen was notably weaker (ΔG = −6.74 kcal·mol^−1^, Ki ≈ 11.4 µM). Key shared contacts include GLN263, ARG266, TRP346, TYR347, PRO350, VAL352, GLY371, TYR373, GLU377 and ARG388, as well as proximity to the heme moiety (HEM550), indicating occupation of the active/heme region; the nearly equivalent binding energies for evodiamine and curcumin suggest comparable complementarity to this site (Figure 7).

**Figure 8:**
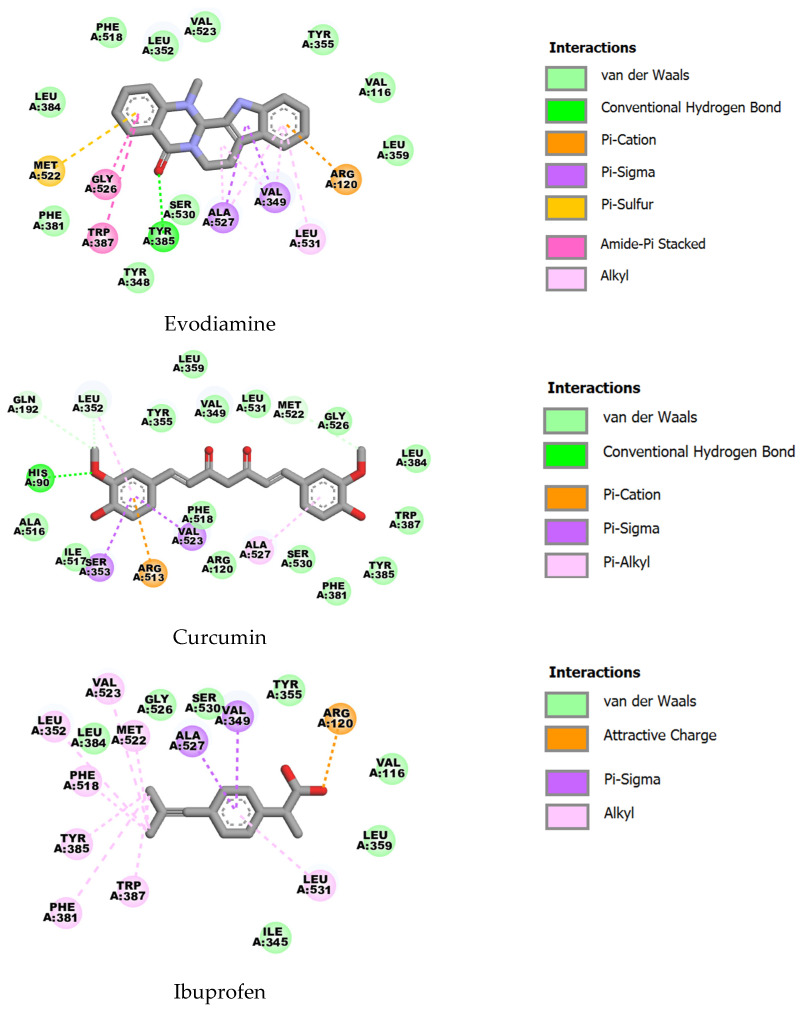
Two-dimensional representations of the binding interactions of evodiamine, curcumin and ibuprofen with COX-2 (PDB ID: 1CX2).

In COX-2 (PDB ID: 1CX2), evodiamine demonstrated the highest predicted affinity (ΔG = −10.02 kcal·mol^−1^, Ki ≈ 0.05 µM), with curcumin also showing strong binding (ΔG = −9.28 kcal·mol^−1^, Ki ≈ 0.16 µM) and ibuprofen substantially weaker (ΔG = −6.52 kcal·mol^−1^, Ki ≈ 16.8 µM). The ligands contact a common set of residues implicated in COX-2 ligand recognition (including ARG120, VAL349, LEU352, TYR355, LEU359, PHE381, LEU384, TYR385, TRP387, MET522, GLY526, ALA527, VAL523, SER530 and LEU531). Evodiamine’s marginally improved energy suggests a tighter fit or additional favorable interactions within the COX-2 binding pocket (Figure 8).

Collectively, these docking results indicate that curcumin and evodiamine possess predicted sub-micromolar to low-micromolar affinities for the studied inflammatory targets, whereas ibuprofen is predicted to bind with substantially lower affinity (tens of micromolar). Many contact residues are conserved among ligands for each target, implying overlapping binding sites; ligand-specific additional contacts and hydrogen-bonding interactions (denoted in the table) likely account for the observed differences in predicted affinity.

The concordance between curcumin’s cellular effects and in silico predictions supports its anti-inflammatory activity in LPS-stimulated RAW264.7 macrophages. Curcumin significantly reduced TNF-α, IL-1β and NO production, and docking predicted high affinity for TNF-α, IL-1β and iNOS. These findings are compatible with two non-mutually exclusive mechanisms: (i) attenuation of pro-inflammatory signaling cascades (e.g., NF-κB and AP-1), resulting in decreased transcription and translation of cytokine genes; and (ii) direct interaction with inflammatory mediators or enzymes (notably iNOS and COX-2), yielding decreased NO and prostanoid synthesis. The predicted binding of curcumin proximal to the iNOS heme pocket is particularly consistent with a potential direct enzymatic inhibition that could underline the observed reduction in NO.

By contrast, evodiamine displayed a clear disconnect between favorable docking predictions and lack of measurable cellular activity at 0.01 µM. Several explanations are plausible: the tested concentration may be below the cellular IC_50_ despite favorable predicted Ki values; evodiamine may have limited cell permeability, rapid intracellular metabolism, or poor accumulation in RAW264.7 cells; substantial protein binding or sequestration in the culture medium could reduce free compound concentration, and binding to soluble cytokines predicted by docking would only be functionally relevant if extracellular occupancy were sufficient to block receptor engagement. Moreover, docking estimates can overpredict binding because they typically omit full solvation effects, protein conformational dynamics, and physiologically relevant multimeric or membrane-associated states.

The observed activity of the evodiamine–curcumin (1:1) mixture at 0.001 µM—an order of magnitude lower than the individual compound concentrations tested—suggests additive or synergistic interactions at sub-micromolar levels. Curcumin is likely the principal contributor to this effect, but evodiamine may potentiate curcumin’s action by enhancing cellular uptake or stability, or by modulating complementary targets such as COX-2. Alternatively, the combination may elicit nonlinear modulation of signaling networks (for example, simultaneous suppression of NF-κB and direct enzymatic inhibition), producing effects not observed with evodiamine alone. These possibilities warrant formal combination–index analysis and mechanistic interrogation.

### 2.5. Relationship Between Molecular Orbitals and Anti-Inflammatory Activities of Evodiamine, Curcumin and Established Anti-Inflammatory Molecule (Ibuprofen)

The highest occupied molecular orbital (HOMO) energy is indicative of a molecule’s propensity to donate electron density (nucleophilicity), whereas the lowest unoccupied molecular orbital (LUMO) energy reflects its propensity to accept electron density (electrophilicity). The HOMO–LUMO gap (ΔE = LUMO − HOMO) serves as a coarse measure of global electronic reactivity and kinetic stability: smaller gaps generally correlate with increased polarizability and chemical reactivity, while larger gaps indicate greater electronic stability and lower nonspecific reactivity.

**Figure 9:**
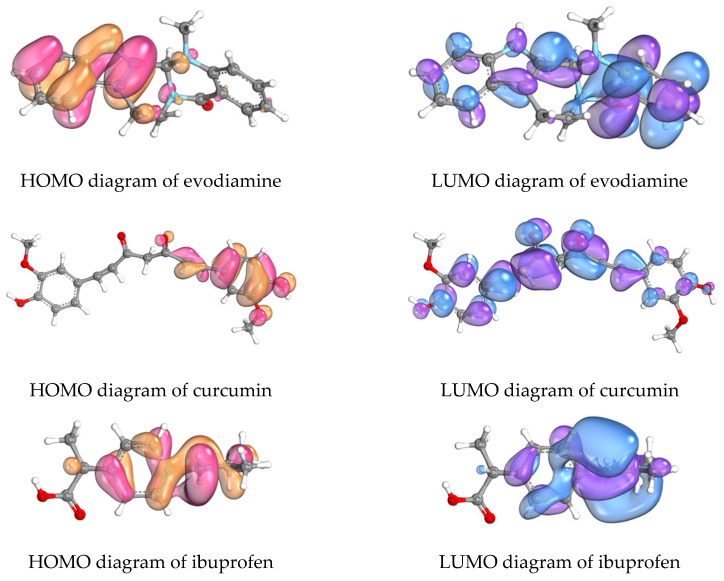
Diagrams depicting the frontier molecular orbitals of evodiamine, curcumin and ibuprofen. The highest occupied molecular orbital (HOMO) is represented in pink and orange shades, while the lowest unoccupied molecular orbital (LUMO) is displayed in violet and blue shades.

As shown in Table 4 and Figure 9, the computed frontier orbital energies are ibuprofen (HOMO = −6.4130 eV; LUMO = 2.2087 eV; ΔE = 8.6217 eV), evodiamine (HOMO = −7.3169 eV; LUMO = 2.3332 eV; ΔE = 9.6501 eV), and curcumin (HOMO = −8.1560 eV; LUMO = 1.7349 eV; ΔE = 9.8909 eV). Ibuprofen exhibits the highest HOMO and smallest ΔE (suggesting relatively greater electronic reactivity), evodiamine occupies an intermediate position, and curcumin displays the lowest LUMO and largest ΔE (indicative of a stronger electron-accepting tendency coupled with greater electronic stability).

Curcumin’s pronounced anti-inflammatory effects (reductions in TNF-α, IL-1β and NO) are consistent with its low LUMO, which may facilitate charge-transfer or polar interactions with protein residues or cofactors in enzymatic active sites (notably iNOS heme-proximal regions identified in docking). Its larger ΔE suggests reduced nonspecific reactivity and may favor sustained, selective binding rather than promiscuous chemical reactivity. By contrast, evodiamine’s higher HOMO and intermediate ΔE imply a greater capacity for electron donation and higher intrinsic reactivity, yet this electronic propensity did not translate into measurable cellular activity at the tested concentration. This discrepancy indicates that frontier orbital energetics alone are insufficient predictors of cellular efficacy, which additionally depends on factors such as membrane permeability, intracellular stability, free (unbound) concentration in culture medium, and kinetics of target engagement. Ibuprofen’s relatively small ΔE and high HOMO illustrate that a favorable electronic profile does not supersede the importance of steric complementarity and specific interaction motifs required for functional inhibition (e.g., its established COX binding mode).

## 3. Discussion

This study demonstrated that curcumin and evodiamine were identified and quantified by HPLC–PDA and LC–MS with high purity (98.04% and 98.08%, respectively). Both compounds modulated inflammatory endpoints in LPS-stimulated RAW264.7 macrophages. The combination treatment produced marked anti-inflammatory effects at a ten-fold lower nominal concentration than curcumin alone (0.001 µM vs. 0.01 µM), consistent with pharmacodynamic potentiation. However, the combination also increased cytotoxicity at higher nominal concentrations, indicating a narrowed therapeutic window that will require careful dose optimization in future studies.

The enhanced cytotoxicity observed at elevated combined concentrations aligns with prior reports of synergistic interactions between curcumin and alkaloids (e.g., piperine), wherein improved bioavailability and complementary pathway modulation augment anticancer activity [27,28,29,30,31]. Mechanistically, such potentiation may reflect (i) inhibition of drug efflux transporters (e.g., P-gp, MRP1, ABCG2) leading to increased intracellular accumulation, and (ii) convergence on pro-death signaling—curcumin’s suppression of NF-κB/mTOR programs together with alkaloid-induced ROS, ER stress, or CHOP/JNK activation—thereby amplifying apoptotic signaling at higher exposures [32,33,34,35,36].

At the cellular outcome level, curcumin consistently suppressed TNF-α, IL-1β and NO, concordant with NF-κB blockade and reduced iNOS transcription. The curcumin–evodiamine combination produced equivalent or greater reductions in these mediators at substantially lower nominal concentrations than curcumin alone, supporting a model of potentiated anti-inflammatory activity despite the combination’s increased cytotoxicity at higher doses. A plausible mechanistic basis for this potentiation is the convergent inhibition of pro-inflammatory signaling: curcumin potently attenuates NF-κB-dependent transcription of COX-2, iNOS, TNF-α and IL-1β [12,37,38], while evodiamine suppresses NF-κB and engages cytoprotective pathways (AKT/Nrf2/HO-1) that can further modulate inflammatory outputs [34,39,40]. Joint NF-κB inhibition and complementary pathway effects provide a rationale for supra-additive suppression of COX-2, iNOS and cytokines when intracellular drug levels permit simultaneous engagement [34,38,41].

For target engagement (docking), strong predicted affinities of curcumin and evodiamine for TNF-α and IL-1β (curcumin particularly favorable) provide a plausible direct mechanism for dampening cytokine signaling or ligand–receptor interactions. Occupation of residues common to known ligand sites indicates these phytochemicals can access functionally relevant pockets. For iNOS, both ligands were predicted to bind near the heme/active region, supporting a mechanistic basis for the observed NO suppression. Evodiamine’s particularly strong predicted binding to COX-2 suggests a complementary mechanism to curcumin for reducing prostanoid synthesis; this may underlie some aspects of the combination’s efficacy and points to multi-target modulation rather than a single dominant interaction [12,21,42]. The in silico data indicate that multi-target engagement (TNF-α/IL-1β blockade combined with iNOS and COX-2 inhibition) could allow lower doses to achieve meaningful anti-inflammatory outcomes, potentially reducing adverse effects associated with single high-dose therapies if cytotoxicity can be managed.

In silico target engagement supports multi-target mechanisms. Docking predicted favorable affinities of curcumin and evodiamine for TNF-α and IL-1β (with curcumin particularly favorable), suggesting potential direct modulation of cytokine signaling or ligand–receptor interactions. Both ligands were predicted to bind proximate to the iNOS heme/active region, consistent with observed NO suppression, and evodiamine showed particularly strong predicted binding to COX-2, indicating a complementary route to reduce prostanoid synthesis. These data are compatible with multi-target engagement (TNF-α/IL-1β plus iNOS and COX-2) as a mechanism enabling lower effective doses and enhanced anti-inflammatory outcomes [12,21,42].

Frontier molecular orbital (FMO) analyses indicate distinct electronic properties that may influence binding modalities: ibuprofen’s higher HOMO and smaller gap suggest greater electron-donating/polarizable character; curcumin’s lower LUMO and larger gap are consistent with electronic stability and selective electron acceptance (favoring H-bonding and π-stacking); evodiamine’s intermediate profile may facilitate complementary hydrophobic and steric interactions within target pockets such as COX-2. Collectively, the FMO patterns support multimodal binding and a mechanistic rationale for additive or synergistic inhibitory effects on cytokine production and enzyme activity [24,25,43,44].

This study is limited by its exclusive use of an in vitro macrophage model (LPS-stimulated RAW264.7 cells), which, while useful for initial screening, does not fully recapitulate myofascial inflammation in vivo. RAW264.7 is an immortalized murine macrophage line with altered signaling relative to primary macrophages, and LPS stimulation models acute endotoxin-driven activation rather than the multifactorial processes (mechanical stress, nerve–muscle interactions, fibroblast and myocyte involvement, extracellular matrix remodeling, and chronic low-grade inflammation) that characterize myofascial pathology. The monoculture format lacks contributions from other resident cell types (muscle cells, fibroblasts, tenocytes, endothelial cells, sensory neurons) and immune subpopulations, as well as tissue architecture, perfusion, metabolism, and pharmacokinetic factors that determine drug distribution, bioavailability, metabolism, and toxicity. Consequently, the observed anti-inflammatory effects and apparent therapeutic windows may not predict efficacy, dose–response relationships, or safety in target tissues. Furthermore, the study did not include experiments on myofascial- or muscle-derived cells or relevant in vivo models; therefore, conclusions regarding myofascial inflammation remain indirect and translational relevance is limited. The apparent synergy of the combination is also insufficiently demonstrated, as only a single mixture ratio and a restricted concentration range were tested; formal combination analyses (e.g., dose–response matrices, combination–index/isobologram or response-surface models) are required to distinguish true synergy from additive or independent effects and to define optimal ratios and therapeutic windows. Another major limitation is the lack of bioavailability and ADME characterization. Curcumin in particular exhibits poor aqueous solubility, limited chemical stability, rapid metabolic conversion, and low systemic exposure, so in vitro activity may not translate to achievable in vivo tissue concentrations. Without physicochemical, metabolic, pharmacokinetic, or formulation data, translational prospects and dose selection remain uncertain. Finally, interpretation of anti-inflammatory readouts is complicated by the combination’s increased cytotoxicity, which could confound reductions in cytokines and nitric oxide through nonspecific cellular impairment rather than target-specific inhibition. Complementary viability and mechanistic assays (apoptosis/necrosis, cell-cycle analysis, pathway profiling) are needed to dissociate specific anti-inflammatory effects from off-target toxicity. Computational results (docking and HOMO–LUMO descriptors) generate testable hypotheses but do not establish binding modes, kinetics, or functional inhibition under physiological conditions; higher-level computational methods (QM/MM, explicit-solvent MD, free-energy calculations) and orthogonal experimental target-engagement assays (biophysical binding, enzymatic inhibition) are warranted.

The next steps include validation in primary human cells and co-culture/3D models, comprehensive combination pharmacology studies, ADME/PK and formulation work to address bioavailability, and mechanistic and in vivo efficacy/toxicity studies to support translation.

## 4. Materials and Methods

### 4.1. Materials

Evodiamine and curcumin raw materials were purchased from MySkinRecepies (Bangkok, Thailand). Chromatography-grade acetonitrile and methanol were obtained from Merck (Darmstadt, Germany). Phosphoric acid was supplied by Sigma-Aldrich Co. (St. Louis, MO, USA). Evodiamine and curcumin reference standards were procured from ChemFaces (Wuhan, Hubei, China). Murine macrophage RAW264.7 cell line (ATCC TIB-71) was purchased from Ameri-can Type Culture Collection (Manassas, VA, USA). Dulbecco’s modified Eagle’s medium (DMEM) high glucose and antibiotic–antimycotic solution were obtained from Gibco (Carlsbad, CA, USA). Fetal bovine serum (FBS), non-US origin, N-(1-Naphthyl) ethylenediamine dihydrochloride, lipopolysaccharide (LPS), and celecoxib were obtained from Sigma-Aldrich (St. Louis, MO, USA). CCK-8 was obtained from Dojindo (Nagasaki, Japan). Dimethyl sulfoxide (DMSO) and phosphoric acid were purchased from Merck (Darmstadt, Germany). Tumor necrosis factor-alpha (TNF-α) and interleukin-1β (IL-1β) ELISA kits were obtained from AbClonal (Woburn, MA, USA). Sulfanilamide was obtained from Panreac (Barcelona, Spain). Sodium nitrite was obtained from Loba Chemie (Mumbai, India).

### 4.2. HPLC Analysis of Evodiamine and Curcumin Raw Materials

The HPLC system used in this study was an Agilent 1260 Infinity II (Santa Clara, CA, USA), comprising a quaternary pump, autosampler, multi-column thermostat, and photodiode array detector. Separation was performed on an ACE C18-AR column (4.6 × 250 mm, 5 µm; Aberdeen, UK) with a Phenomenex C18 guard column (4 × 3 mm, 5 µm; Torrance, CA, USA). The mobile phase comprised 0.1% phosphoric acid in ultrapure water (phase A), acetonitrile (phase B), and methanol (phase C). Sample analysis employed an isocratic elution of 40% A, 40% B, and 20% C at a flow rate of 1.0 mL·min^−1^. Detection wavelengths were 226 nm for evodiamine and 426 nm for curcumin. The column temperature was maintained at 25 °C and the injection volume was 10 µL. The analytical method was subjected to system suitability testing and validated in accordance with ICH guidelines and AOAC recommendations (2016).

### 4.3. In Vitro Anti-Inflammatory Activity Assay

#### 4.3.1. Cell Culture

RAW264.7 cells were cultured in high-glucose Dulbecco’s modified Eagle’s medium (DMEM) supplemented with 10% fetal bovine serum and 1% antibiotic–antimycotic solution, maintained at 37 °C in a humidified atmosphere containing 5% CO_2_. Monolayer cultures were kept in exponential growth by subculturing every 3–4 days.

#### 4.3.2. Cell Viability Assay

Cell viability was assessed using the CCK-8 assay. RAW264.7 cells were seeded at 1 × 10^5^ cells/mL (100 µL/well) in 96-well plates. After 24 h, the growth medium (high-glucose DMEM with 10% FBS and 1% antibiotic-antimycotic) was replaced with medium containing evodiamine or curcumin dissolved in 0.5% DMSO to give final concentrations of 0.001, 0.01, 0.1, 1, and 10 µM. Medium with 0.5% DMSO served as the vehicle control. Cells were incubated for 24 h at 37 °C, after which the medium was removed and 100 µL of DMEM (without supplements) plus 10 µL CCK-8 reagent were added to each well. Following a 2 h incubation at 37 °C, absorbance was read at 450 nm. Results are presented as mean ± SD (*n* = 3, each run in triplicate), with untreated cells cultured in complete medium defined as 100% viability. Percent viability was calculated accordingly.

% cell viability = (OD of treated cell/OD of untreated cell) × 100(1)

The concentration of quercetin that showed cell viability greater than 80% was further used to evaluate its anti-inflammation activity via TNF-α, IL-1β and NO secretions.

#### 4.3.3. Anti-Inflammation Assay

RAW264.7 cells were seeded at 5 × 10^5^ cells/mL (1000 µL/well) in 24-well plates. After 24 h, complete medium (high-glucose DMEM with 10% FBS and 1% antibiotic-antimycotic) was replaced with complete medium containing quercetin dissolved in 0.5% DMSO at concentrations that preserved >80% cell viability. Medium containing 0.5% DMSO served as the vehicle control; 25 µM celecoxib was used as a positive control. Cells were pretreated for 1 h at 37 °C, then stimulated with lipopolysaccharide (LPS) to a final concentration of 1 µg·mL^−1^ and incubated for 24 h at 37 °C (*n* = 3). After incubation, supernatants were collected and stored at −80 °C until analysis of TNF-α, IL-1β, and NO.

##### TNF-α and IL-1β Secretion Assay Using ELISA Kit

TNF-α and IL-1β concentrations in culture supernatants were measured using AbClonal ELISA kits according to the manufacturer’s protocol. Briefly, 100 µL of appropriately diluted samples or TNF-α/IL-1β standards were added to mouse TNF-α- or IL-1β-antibody precoated 96-well plates and incubated at 37 °C for 2 h. Wells were emptied and 100 µL of biotin-conjugated detection antibody was added and incubated at 37 °C for 1 h. After washing, 100 µL of streptavidin-HRP was added and incubated at 37 °C for 30 min. Plates were washed again, 100 µL TMB substrate was added and incubated at 37 °C for 15 min, then the reaction was stopped with 50 µL stop solution. Absorbance was read at 450 and 570 nm.

##### Nitric Oxide Assay

The collected supernatants were assayed for nitric oxide using the Griess reaction and read at 540 nm. Nitrite concentrations were determined from a sodium nitrite standard curve. Briefly, 100 µL of each supernatant was placed in a 96-well plate, followed by 100 µL Griess reagent (1% sulfanilamide and 0.1% N-1-naphthylethylenediamine dihydrochloride in 2.5% phosphoric acid); the mixture was incubated for 10 min, and absorbance was measured.

### 4.4. Molecular Docking Analysis of Evodiamine, Curcumin and Ibuprofen with TNF-α, IL-1β, Inos and COX-2

Crystal structures of TNF-α (PDB ID: 2AZ5) [45], IL-1β (PDB ID: 5R8Q) [46], iNOS (PDB ID: 4CX7) [47], and COX-2 (PDB ID: 1CX2) [48] were retrieved from the Protein Data Bank. Prior to export to .pdb format, nonessential solvent molecules and bound ligands were removed. Ligand structures were initially drafted in Avogadro v1.2.0 and converted to three-dimensional .pdb files. Molecular docking was performed with AutoDock 4.2 [49]. Input files were prepared in AutoDockTools v1.5.6 (The Scripps Research Institute) by adjusting atom types, deleting waters, and adding polar hydrogens and Gasteiger charges. Grid boxes (40 × 40 × 40 grid points, default spacing) were centered at coordinates −19.163, 74.452, 33.837 (2AZ5); 39.787, 1.608, 73.990 (5R8Q); −14.511, −64.279, 17.811 (4CX7); and 23.947, 21.582, 15.436 (1CX2). Prepared structures were saved in PDBQT format and docked using AutoDock 4.2; the poses with the lowest binding energies were retained for further analysis. Docking validation was performed by redocking the co-crystallized ligand and calculating root-mean-square deviation (RMSD), with RMSD ≤ 2.0 Å considered acceptable. Visual inspection and presentation of docked complexes were carried out in Discovery Studio (BIOVIA, Dassault Systèmes). Binding free energy (ΔG) and estimated inhibition constant (Ki) were used to evaluate the most favorable ligand poses, using the same docking parameters applied to the native ligands.

### 4.5. Calculation of Frontier Molecular Orbitals and Visualization

Avogadro (v. 1.2.0) was employed to construct the initial molecular geometries [50]. The resulting optimized Cartesian (xyz) coordinates were exported and used to generate Orca input (.inp) files, with calculations specified at the restricted Hartree–Fock (RHF) level using the def2-SVP basis set. Orca [51] was then executed on these input files by opening an elevated command prompt, navigating to the folder containing the .inp files, and invoking the Orca executable (assuming Orca had been added to the system PATH) using the appropriate command.


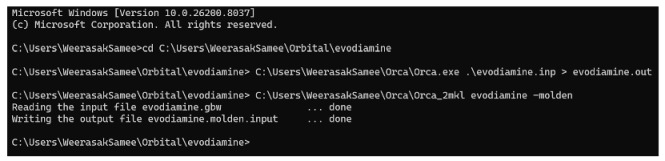


Molden output files were visualized using IboView (v. 20211019) [52], which was utilized to perform intrinsic bond orbital (IBO) computations and to examine molecular geometries and molecular orbital (MO) visualizations.

### 4.6. Statistical Analysis

All data were expressed as mean ± standard deviation (SD) from at least three independent experiments. Statistical analysis was conducted using GraphPad Prism version 8.0 (GraphPad Software, San Diego, CA, USA). One-way ANOVA followed by Tukey’s post hoc test was used for multiple group comparisons, while unpaired *t*-tests were used for two-group comparisons. A *p*-value of <0.05 was considered statistically significant.

## 5. Conclusions

The combined in vitro and in silico dataset supports that curcumin and evodiamine are promising complementary anti-inflammatory agents that engage multiple inflammation mediators (TNF-α, IL-1β, iNOS, COX-2) and that their distinct electronic and structural properties may underlie additive or synergistic activity. Curcumin appears to be the principal driver of cytokine and NO suppression at the tested doses, while evodiamine contributes notable predicted COX-2 engagement and, together with curcumin, offers multi-target coverage. Translation toward therapeutic development for myofascial inflammation will require rigorous dose-optimization, mechanistic validation of direct target inhibition, cytotoxicity mitigation, and in vivo efficacy and safety studies.

## Figures and Tables

**Table 1:** Identification and quantification of evodiamine and curcumin in raw materials.

Reference Standards	Retention Time of Reference Standards (Minutes)	Retention Time of Raw Materials (Minutes)	% Purity (*n* = 3)
Evodiamine	8.61	8.60	98.08 ± 1.92
Curcumin	9.53	9.52	98.04 ± 1.86

**Table 2:** TNF-α, IL-1β and NO levels in LPS-stimulated RAW 264.7 cells.

Sample	TNF-α (ng·mL^−1^)		IL-1β (pg·mL^−1^)		NO (µM)	
	Mean	SD	Mean	SD	Mean	SD
0.5% DMSO (solvent control)	1.99 *	0.19	0.00	0.00	10.00	0.99
1 µg·mL^−1^ LPS (negative control)	4.81	0.90	6.12	9.42	13.77	0.62
50 µM Ibuprofen (positive control)	3.91 *	0.37	0.00	0.00	6.33 *	0.36
Evodiamine 0.01 µM	5.56	0.18	2.68	4.22	15.18	0.28
Curcumin 0.01 µM	3.60 *	0.28	0.00	0.00	7.24 *	0.99
Mixture of evodiamine and curcumin in a 1:1 ratio 0.001 µM	3.12*	0.38	0.00	0.00	6.24 *	0.74

* *p* < 0.05 compared with 1 µg·mL^−1^ LPS-stimulated RAW 264.7 cells (negative control).

**Table 3:** Results of molecular docking studies of evodiamine, curcumin and ibuprofen with TNF-α, IL-1β, iNOS, and COX-2 targets.

Ligand	Target	Binding Energy (Kcal/mole)	Ki (µM)	Contacted Amino Acid *
Evodiamine	TNF-α (PDB ID: 2AZ5)	–8.35	0.76	LEU_A57, TYR_A59, SER_A60, GLY_A61, TYR_A119, LEU_A120, GLY_A121, TYR_A151, TYR_B59, SER_B60, TYR_B119, TYR_B151,
Curcumin		–10.18	0.03	TYR_A59, SER_A60, GLY_A61, TYR_A119, LEU_A120, GLY_A121, GLY_A122, TYR_A151, TYR_B59, GLN_B61, TYR_B119, LEU_B120, GLY_B121, TYR_B151
Ibuprofen		–6.00	39.70	TYR_A59, SER_A60, GLY_A61, TYR_A119, LEU_A120, GLY_A121, TYR_A151, TYR_B59, SER_B60, GLN_B61, TYR_B119, LEU_B120, GLY_B121, TYR_B151
Evodiamine	IL-1β (PDB ID: 5R8Q)	−7.72	2.19	TYR24, GLU25, LEU26, LEU69, LYS74, THR79, LEU80, GLN81, LEU82, PRO131, VAL132
Curcumin		−8.73	0.40	TYR24, GLU25, LEU26, LEU69, LYS74, THR79, LEU80, GLN81, LEU82, PRO131, VAL132, PHE133, LEU134
Ibuprofen		–6.24	26.87	TYR24, GLU25, LEU26, LEU69, LYS74, THR79, LEU80, GLN81, LEU82, VAL132
Evodiamine	iNOS (PDB ID: 4CX7)	−8.95	0.27	GLN263, ARG266, TRP346, TYR347, PRO350, VAL352, PHE369, GLY371, TYR373, GLU377, ASP382, ARG388, HEM550
Curcumin		−8.87	0.31	GLN263, ARG266, TRP346, TYR347, PRO350, VAL352, GLY371, TRP372, TYR373, MET374, GLU377, ARG381, ARG388, PRO467, HEM550
Ibuprofen		–6.74	11.40	GLN263, ARG266, TRP346, TYR347, PRO350, VAL352, GLY371, TRP372, TYR373, MET374, GLU377, ARG381, ARG388, PRO467, HEM550
Evodiamine	COX-2 PDB ID: 1CX2	−10.02	0.05	VAL116, ARG120, TYR347, VAL349, ALA351, LEU352, TYR355, LEU359, PHE369, PHE381, LEU384, TYR385, TRP387, MET522, GLY526, ALA527, VAL523, SER530, LEU531, PHE581
Curcumin		−9.28	0.16	HIS90, ARG120, GLY192, VAL349, LEU352, SER353, TYR355, LEU359, PHE381, LEU384, TYR385, TRP387, MET522, GLY526, ARG513, ALA516, ILE517, PHE519, ALA527, VAL523, SER530, LEU531,
Ibuprofen		–6.52	16.76	VAL116, ARG120, ILE345, VAL349, LEU352, TYR355, LEU359, PHE381, LEU384, TYR385, TRP387, PHE518, MET522, GLY526, ALA527, VAL523, SER530, LEU511

* The green color represents the amino acids involved in hydrogen bonding.

**Table 4:** Calculated HOMO and LUMO energies (in electron volts, eV) of evodiamine, curcumin and ibuprofen.

Compounds	HOMO (eV)	LUMO (eV)	ΔE (eV)
Evodiamine	–7.3169	2.3332	9.6501
Curcumin	–8.1560	1.7349	9.8909
Ibuprofen	−6.4130	2.2087	8.6217

## Data Availability

The original contributions presented in this study are included in the article. Further inquiries can be directed to the corresponding author.

## Abbreviations

The following abbreviations are used in this manuscript:

ABCG2	ATP-binding cassette sub-family G member 2
AKT	Protein kinase B
AOAC	Association of Official Analytical Collaboration
°C	Celsius degree
CHOP	C/EBP (CCAAT/enhancer-binding protein) homologous protein
cm	Centimeter
COX-2	Cyclooxygenase-2
2D	Two-dimension
DAMPs	Damage-associated molecular patterns
DMSO	Dimethyl sulfoxide
ER	Endoplasmic reticulum
eV	Electron volt
HO-1	Heme oxygenase-1
HOMO	Highest occupied molecular orbital
HPLC	High Performance Liquid Chromatography
IBO	Intrinsic bond orbital
IC_50_	Half–maximal inhibitory concentration
ICH	The International Council for Harmonisation of Technical Requirements for Pharmaceuticals for Human Use
IKK	IκB kinase
iNOS	Inducible nitric oxide synthase
JNK	c-Jun N-terminal kinase
IL-1β	Interleukin-1 beta
L	Litter
LC-MS	Liquid chromatography-Mass spectrometry
LPS	Lipopolysaccharide
LUMO	Lowest unoccupied molecular orbital
M	Mass
MD	Molecular dynamics
mL	Milliliter
MM	Molecular mechanics
Mol	Mole
MRP1	Multidrug resistance-associated protein 1
mTOR	Mechanistic (or mammalian) target of rapamycin
MW	Molecular weight
*m*/*z*	Mass per charge
NF-κB	Nuclear factor kappa-light-chain-enhancer of activated B cells
Nrf2	Nuclear factor erythroid 2–related factor 2
PBD	Protein data bank
P-gp	P-glycoprotein
PDA	Photo diode array
QM	Quantum mechanics
ROS	Reactive oxygen species
RT	Retention time
SD	Standard deviation
TNF-α	Tumor necrosis factor-alpha
UV	Ultraviolet
VIS	Visible
ΔE	The difference between HOMO and LUMO energies
ΔG	Gibbs free energy
µg	Micro gram
µM	Micro molar
%RSD	Percentage relative standard deviation
